# Five Proteins of *Laodelphax striatellus* Are Potentially Involved in the Interactions between Rice Stripe Virus and Vector

**DOI:** 10.1371/journal.pone.0026585

**Published:** 2011-10-20

**Authors:** Shuo Li, Ruyi Xiong, Xifeng Wang, Yijun Zhou

**Affiliations:** 1 Department of Plant Pathology, College of Plant Protection, Nanjing Agricultural University, Key Laboratory of Monitoring and Management of Crop Diseases and Pest Insects, Ministry of Agriculture, Nanjing, People's Republic of China; 2 Institute of Plant Protection, Jiangsu Academy of Agricultural Sciences, Jiangsu Technical Service Center of Diagnosis and Detection for Plant Virus Diseases, Nanjing, People's Republic of China; 3 State Key Laboratory for Biology of Plant Diseases and Insect Pests, Institute of Plant Protection, Chinese Academy of Agricultural Sciences, Beijing, People's Republic of China; University of South Florida College of Medicine, United States of America

## Abstract

Rice stripe virus (RSV) is the type member of the genus *Tenuivirus*, which relies on the small brown planthopper (*Laodelphax striatellus* Fallén) for its transmission in a persistent, circulative-propagative manner. To be transmitted, virus must cross the midgut and salivary glands epithelial barriers in a transcytosis mechanism where vector receptors interact with virions, and as propagative virus, RSV need utilize host components to complete viral propagation in vector cells. At present, these mechanisms remain unknown. In this paper, we screened *L. striatellus* proteins, separated by two-dimensional electrophoresis (2-DE), as potential RSV binding molecules using a virus overlay assay of protein blots. The results, five *L. striatellus* proteins that bound to purified RSV particles in vitro were resolved and identified using mass spectrometry. The virus-binding capacities of five proteins were further elucidated in yeast two-hybrid screen (YTHS) and virus-binding experiments of expressed proteins. Among five proteins, the receptor for activated protein kinase C (RACK) and glyceraldehyde-3-phosphate dehydrogenase (GAPDH3) did not interact with RSV nucleocapsid protein (NCP) in YTHS and in far-Western blot, and three ribosomal proteins (RPL5, RPL7a and RPL8) had specific interactions with RSV. In dot immunobinding assay (DIBA), all five proteins were able to bind to RSV particles. The five proteins' potential contributions to the interactions between RSV and *L. striatellus* were discussed. We proposed that RACK and GAPDH3 might be involved in the epithelial transcytosis of virus particles, and three ribosomal proteins probably played potential crucial roles in the infection and propagation of RSV in vector cells.

## Introduction

Rice stripe virus (RSV), the type member of the genus *Tenuivirus*, was first reported in Japan in 1975 [1] and is currently present in many East Asian countries, including China. RSV has been reported to cause severe disease in rice fields in China in recent years [2].

RSV is known to be transmitted mainly by the small brown planthopper (*Laodelphax striatellus* Fallén) in a persistent, circulative-propagative manner [3]. After invaded into *L. striatellus*, RSV can escape from midgut, salivary gland and ovary barriers and propagate in the body, since evidences have revealed that amorphous or filamentous inclusions of RSV exists in the cytoplasm of midgut epithelial cells, salivary gland as well as fat body [4], [5]. Female and male adults, nymphs all can transmit virus, while *L. striatellus* nymphs were reported as more efficient vectors than adults, and females as more efficient vectors than males for RSV transmission [3]. Moreover, it has been confirmed that RSV particles exist in follicular cells of the ovarioles and can be transmitted from female adults to their progeny via eggs [5]. The epidemic and outbreak of rice stripe disease have close relationship with the outbreak of viruliferous populations of *L. striatellus*. Even at a lower density, viruliferous vectors could lead to significant yield losses by virus infection [6]. Therefore, it is crucial for disease control to research the mechanisms how RSV is transmitted specifically by *L. striatellus*. At present, the molecular interaction mechanisms between RSV and vector remain unclear.

Particles of RSV are filamentous, 3–8 nm in diameter and 500–2000 nm in length [7], [8]. Tenuiviruses have phylogenetic similarity to some members of *Bunyaviridae* [3], [9]. Thus, RSV might be expected to have the enveloped virion form of Bunyaviruses, but no such form has so far been found in either infected plants or insects [5]. Therefore, it is suggested that the viral determinant of transmission specificity may be localized on the capsid of RSV, which is composed of the nucleocapsid proteins (NCP) of 35 kDa.

The movement and replication of the persistent-propagative viruses in the insect vectors require specific interactions between virus and vector components [10]. The specific receptors of RSV particles in *L. striatellus* remain to be determined. A common strategy to study vector components that interact with virus particles is based on the far-Western blot methodology. The far-Western blot, also known as virus overlay assay, has been used successfully to detect proteins that are potential virus receptors in the body of insect vectors. Initially, several proteins within *Myzus persicae* were found to bind to Potato leafroll virus (PLRV) in vitro [11]. Since then, many vector proteins interacting with virus particles were determined. The 50-kDa and 94-kDa proteins of the thrips vector, *Frankliniella occidentalis*, have been certified to bind in vitro to particles of Tomato spotted wilt virus (TSWV). The 94-kDa protein may represent a receptor protein involved in virus circulation through the vector, but probably not in viral uptake in the midgut [12], whereas the 50-kDa protein has been localized in the midgut brush border of thrips [13]. Zhou *et al.* showed the existence of a 32-kDa membrane protein, a potential receptor of Rice ragged stunt virus (RRSV) in the leafhopper *Nilaparvata lugens* [14]. Li *et al.* showed that two proteins, SaM35 and SaM50, in the vector aphid *Sitobion avenae*, could bind specifically to purified Barley yellow dwarf virus MAV (BYDV-MAV) particles, and considered them as potential accessory salivary glands (ASG)-borne receptors for BYDV-MAV in *S. avenae* [15].

In this paper, specific molecular interactions between RSV particles and proteins of *L. striatellus* were investigated, using virus overlay assay as previously used to characterize virus-vector interactions. Our results showed that five proteins of *L. striatellus* bound specifically to purified RSV particles in vitro. These proteins were identified using mass spectrometry, and their virus-binding capacities were further evaluated.

## Results

### Specific binding of RSV particles to *L. striatellus* proteins separated by 2-DE

**Figure 1:**
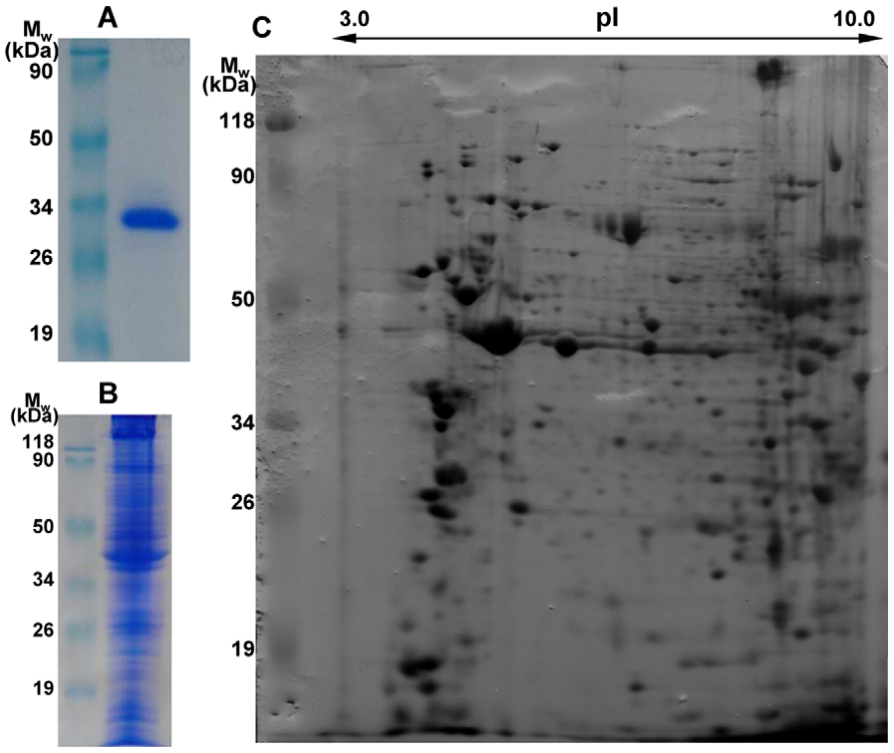
Characterization of purified RSV particles and total proteins from non-viruliferous *L. striatellus*. (A) Purified RSV particles. (B) Separation of *L. striatellus* proteins by 12% SDS-PAGE. (C) Separation of *L. striatellus* proteins by 2-DE. Relative molecular weight markers (M_w_) were indicated on the left and pI ladder on the top.

**Figure 2:**
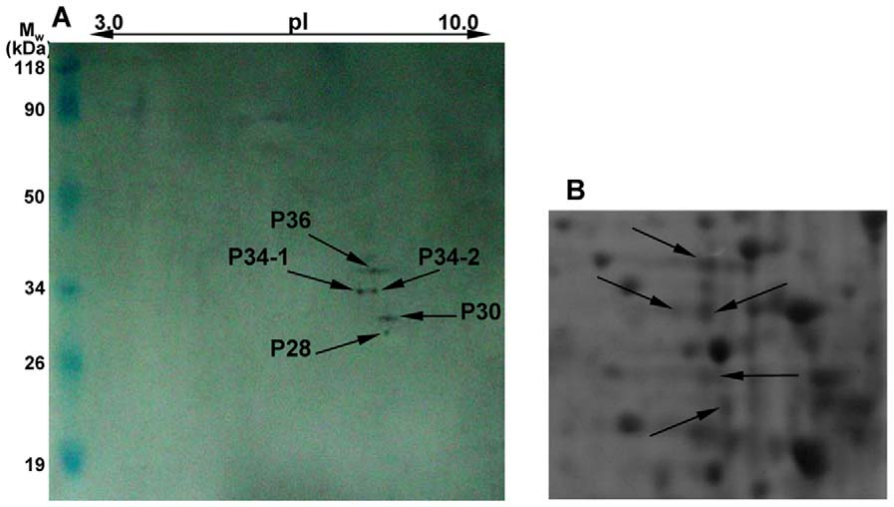
Binding experiment of *L. striatellus* proteins with purified RSV particles. (A) Binding experiment. M_w_ was indicated on the left and pI ladder on the top. (B) Selected area of 2-DE gels showing *L. striatellus* proteins that bound in vitro to RSV particles. 2D spots indicated by arrows were selected for the LC-MS-MS analysis.

In the study, a virus overlay assay was applied to ascertain whether specific binding of RSV particles to immobilized *L. striatellus* proteins would occur. Purified virus particles and the whole-body extracts of high-affinity *L. striatellus* adults (non-viruliferous) were characterized respectively by 12% SDS-PAGE (Fig. 1A and 1B). Insect total proteins, homogenized in IEF sample buffer, were separated by two-dimensional electrophoresis (2-DE) (Fig. 1C). After 2-DE, far-Western blot experiment was carried out. The proteins were blotted onto PVDF membrane and probed with purified RSV. As shown in Fig. 2A, RSV particles were able to bind specifically to five proteins: P36, P34-1, P34-2, P30 and P28, all with basic isoelectric points (pI).

### Identification of the nature of *L. striatellus* proteins that link RSV particles

Comparisons between the stained electrophoretic profiles of *L. striatellus* proteins in the gel and results of far-Western blot experiments on the membrane allowed the unambiguous selection of protein spots from 2-DE gels (indicated by arrows in Fig. 2B) for Nano LC-ESI-CID-MS/MS analysis. Selected protein spots were subjected to in-gel trypsin digestion. Generated peptides were analyzed by LC-MS/MS on an LTQ Orbitrap linear ion trap mass spectrometer connected with an Agilent 1100 HPLC system. Identified proteins were listed in Table 1, including the receptor for activated protein kinase C (RACK), 60 S ribosomal protein (r-protein) L5 (RPL5), glyceraldehyde-3-phosphate dehydrogenase (GAPDH3), 60 S r-protein L7a (RPL7a), 60 S r-protein L8 (RPL8). The experimentally observed relative molecular weights and pI values were in good agreement with the calculated values reported in the NCBI database for these five proteins.

**Table 1:** Identification of *L. striatellus* proteins using Nano LC-ESI-CID-MS/MS.

Spots	Identified proteins	Score	Coverage(%)	NCBI code	NCBIcalculated*M*_w_(kDa)	Experimentallyderived valuesfor *M*_w_(kDa)
P36	RACK (*Tribolium castaneum*)	409	30%	gi|91089633	36.171	36.0
P34-1	60S RPL5 (*Lysiphlebus testaceipes*)	107	6%	gi|74829222	34.621	34.0
P34-2	GAPDH3 (*Homalodisca coagulata*)	492	30%	gi|46561740	35.568	34.0
P30	60S RPL7a (*Macaca mulatta*)	247	14%	gi|109102044	30.128	30.0
P28	60S RPL8 (*Triatoma infestans*)	338	17%	gi|149689084	28.276	28.0

Selected protein spots were subjected to in-gel trypsin digestion. Protein fragments were then analyzed by LC-MS/MS on an LTQ Orbitrap linear ion trap mass spectrometer connected with an Agilent 1100 HPLC system. Data analyses were done with Bioworks Browser software and Mascot against NCBInr database. The score is significant if it is higher than 39.

### Cloning and sequencing of virus-binding proteins' ORFs

**Figure 3:**
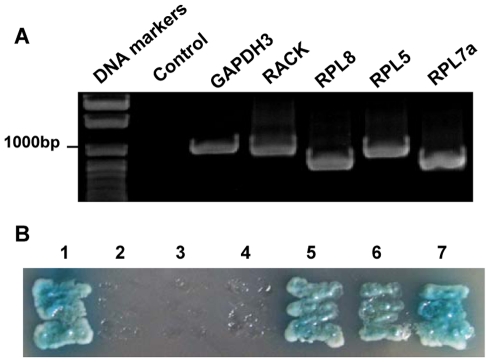
RT-PCR analysis and yeast two-hybrid screen of five virus-binding proteins. (A) Electrophoresis of gene amplified products was in 1% agarose gel. DNA markers were indicated on the left and genes on the top. (B) Yeast two-hybrid assay of protein-protein interactions between RSV-NCP and five virus-binding proteins (GAPDH3, RACK, RPL8, RPL5 and RPL7a). Yeast cotransformants were incubated on the selective medium (SD/–Ade/–His/–Leu/–Trp plus X-α-Gal) at 28°C for 4 d. 1: positive control; 2: negative control; 3: GAPDH3 & RSV-NCP; 4: RACK & RSV-NCP; 5: RPL8 & RSV-NCP; 6: RPL5 & RSV-NCP; 7: RPL7a & RSV-NCP.

To further study interactions between virus-binding proteins and RSV, we cloned their full-length genes. The peptide sequences from LC-MS/MS (listed in Table 2) were used to search matched proteins against the *L. striatellus* transcriptome database with the tBlastn tool. After obtaining contigs, five pairs of primers for specific amplification of virus-binding proteins' ORFs were designed according to respective contig sequences. Single amplified products of five genes were obtained via RT-PCR (Fig. 3A), cloned and sequenced. The sequencing results indicated that the sequences of five full-length ORFs were identical to respective contig sequences. The *L. striatellus* RACK, RPL5, GAPDH3, RPL7a and RPL8 genes (GenBank accession numbers: HQ385972, HQ385973, HQ385974, HQ385975, HQ385976) were respectively 948 bp, 900 bp, 999 bp, 807 bp and 777 bp.

**Table 2:** The peptide sequence tag from LC-MS/MS.

proteins	peptide sequences
RACK	**DKTLIIWK** **LWDLAAGR** **DVLSVAFSVDNR** **LWNTLAECK** **FSPNHANPIIVSAGWDR** **VWNLTNCR** **INHSGHTGYLNTVTVSPDGSLCASGGK** **AMLWDLNDGK** **HLHTLDHNDIITALCFSPNR** **YWLCAAFGPSIK** **IWDLETK**
RPL5	**TDYYAR** **RLIVQDK** **YNTPK** **DITCQIAYSR** **IEGDKIVCAAYSHELPK** **IVCAAYSHELPK** **VGLTNYASAYCTGLLLAR** **VFGAMK** **GAVDGGLNIPHSVK** **AHEAIR** **KLTLSER** **AGYFK**
GAPDH3	**FKGEVK** **AIPWGK** **IVSNASCTTNCLAPLAK** **VIHDNFEIVEGLMTTVHATTATQK** **GAQQNIIPAATGAAK** **LTGMAFR** **VPVPNVSVVDLTVR** **AASEGPLK** **LISWYDNEFGYSSR** **VIDLIK**
RPL7a	**KVAAAPLVVK** **KVVNPLFEK** **NFGIGQDIQPK** **QTATQLFK** **QGVNTVVK** **MGVPYCIIK** **KTCTCLALTNVESGDR** **LVEAVK** **HWGGGLLGSK**
RPL8	**LRPLDYAER** **GAPLAVVHFR** **ASGNYATVIAHNPDTK** **AMVGIVAGGGR** **VGLIAAR**

### Interactions between RSV-NCP and the virus-binding proteins in the yeast two-hybrid system

To determine whether virus-binding proteins interact with the viral nucleocapsid protein, RSV-NCP gene was fused in-frame to the GAL4 transcriptional activation domain (AD) in pGADT7 vector, while each of five potential genes was fused in-frame to the GAL4 DNA-binding domain (BD) in pGBKT7 vector. The plasmids containing five potential genes were cotransformed respectively with pGAD-NCP into yeast cells. Yeast cells cotransformed with plasmids pGBKT7-53/pGADT7-RecT and pGBKT7-Lam/pGADT7-RecT served as positive and negative controls. The results showed that yeast cells cotransformed with pGBK-RPL5/pGAD-NCP, pGBK-RPL7a/pGAD-NCP and pGBK-RPL8/pGAD-NCP as positive controls as were able to grow on selective medium (SD/–Ade/–His/–Leu/–Trp supplemented with X-α-gal) (Fig. 3B), which indicated that specific interactions occurred between three proteins (RPL5, RPL7a and RPL8) of *L. striatellus* and RSV-NCP in the yeast two-hybrid system.

### Binding of expressed five *L. striatellus* proteins to RSV particles in vitro

**Figure 4:**
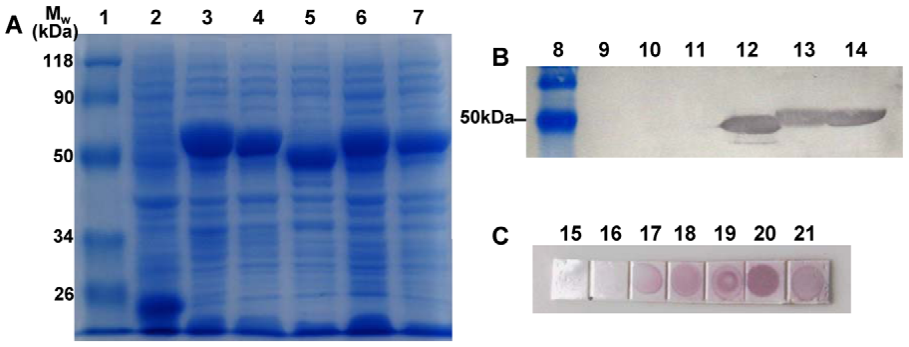
SDS-PAGE analysis and binding experiments with RSV particles of expression products of virus-binding proteins. (A) Expression products were separated by 12% SDS-PAGE. (B) Binding experiments with purified RSV particles by far-Western blot. (C) Binding experiments with RSV particles by DIBA. Lane 1, 8: relative molecular weight markers; lane 2, 9, 16: pET32a vector; lane 3, 10, 17: pET-GAPDH3; lane 4, 11, 18: pET-RACK; lane 5, 12, 19: pET-RPL8; lane 6, 13, 20: pET-RPL5; lane 7, 14, 21: pET-RPL7a; lane 15: *E. coli* BL21 (wt).

To detect binding ability of screening *L. striatellus* proteins to RSV particles in vitro, RACK, RPL5, GAPDH3, RPL7a and RPL8 proteins were expressed in *E. coli* BL21 (DE3) pLys S cells using pET32a vector. The expression products were evaluated by 12% SDS-PAGE (Fig. 4A). The results demonstrated that five *L. striatellus* proteins expressed in *E. coli* had the expected size of 54.5 kDa, 53 kDa, 54.5 kDa, 49.5 kDa, and 47 kDa, respectively. Expression products were bloted onto the PVDF membrane and renatured. Far-Western blot indicated that three protein bands (RPL5, RPL7a and RPL8) were able to bind specifically to virus particles (Fig. 4B). DIBA indicated that all five *L. striatellus* proteins had ability to bind to RSV particles (Fig. 4C).

## Discussion

Insect transmission of plant viruses involves complex interactions between viral proteins and vector-associated compounds [16], [17]. The interactions between plant viruses and their vectors are crucial to the transmission of virus. To research interaction mechanisms is foundation of developing new technologies for virus disease prevention and control, which will offer novel opportunities for disease control, so some researchers have focused on molecular aspects of these interactions. Recent investigations have resulted in a better elucidation on the replication sites, overcoming transmission barriers and tissue tropism in insect vectors of several propagative viruses, including TSWV (*Bunyaviridae*), Rice dwarf virus (RDV, *Reoviridae*), Wound tumor virus (WTV, *Reoviridae*) [10], but little is known about *L. striatellus* contribution to RSV transmission. In this study, using virus overlay assay and mass spectrometry, we identified five unexpected *L. striatellus* proteins that bound to RSV particles in vitro, including RACK, GAPDH3, RPL5, RPL7a and RPL8.

P36, which bound to RSV particles, was determined as RACK. In animals, RACK, as a multifunctional, WD40 motif-containing protein, is important in regulating several cell surface receptors and intracellular protein kinases [18]. RACK, a homolog of the β subunit of G proteins, binds to activated protein kinase C (PKC), acting as an intracellular receptor to anchor the activated PKC to the cytoskeleton and to target the enzyme to the membrane [19]–[21]. In addition to its role as a major signaling event in the cell, PKC can regulate virus endocytic trafficking by phosphorylation [22]. RACK may belong to a membrane complex, and it is a scaffold protein that physically connects various signal transduction components into stable complexes [23] and directs “cross-pathway control” by integrating communication from different signaling pathways (such as Ser/Thr kinases A and C and cAMP signaling cascades) [20], [24]. RACK is localized at internal leaflet of membranes and on cytoskeleton elements in the cytosol [19], [21]. RACK is known to interact with β subunits of integrins [25], which are components of the extracellular matrix basal lamella of invertebrates including insects [26], while integrins can interact with viruses [27]. RACK of the vector aphid *M. persicae* can bind to wild-type Beet western yellows virus (BWYV, *Polerovirus*), but not to the two mutants with low efficiency in endocytosis/transcytosis, which suggests that RACK plays a key role in this process [28].

P34-2 was determined as the GAPDH3. In animals, GAPDH3, besides playing an important role in glycolysis in the cytosol, has diverse cellular functions depending on its localization in each subcellular compartment [29]. When being phosphorylated by PKC [30], GAPDH3 binds to membranes and regulates endocytosis by probably promoting formation of microtubule network [29], [31] and exocytosis by catalyzing membrane fusion activity [32]. GAPDH3 is sometimes accessible at cell surface [33]–[35] and binds to fibronectin [33], which is a component of basal lamella of invertebrate cells [26]. GAPDH3 of *M. persicae* has been found to bind to BWYV-wt. It is remarkable that BWYV-wt can bind to GAPDH3 from membrane protein fraction, but not to GAPDH3 from soluble protein fraction, which is probably attributed to a different conformation of GAPDH3 in the membrane compared to its cytosol form [28].

In our experiments, RACK and GAPDH3 from natural insect extraction had ability to bind to RSV particles, and the result of DIBA was also the case, but they had no interaction with RSV-NCP in YTHS and far-Western blot. One explanation was based on that the protein's interaction with virus might rely on their specific conformation. 3-dimensional conformation of RACK and GAPDH3 might be destroyed in YTHS due to fusion proteins' misfolding and in far-Western due to SDS-PAGE denaturation. Due to its localization at the inner membrane leaflet, RACK was clearly not the RSV extracellular receptor, but the binding between RSV particles and RACK suggested that RACK might be a key element of RSV transcytosis mechanism and in direct contact with the virus at some stage of the process. We hypothesized that GAPDH3 could be part of the RSV receptor in *L. striatellus* if localized at the outside leaflet of the plasmalemma or in the basal lamella of midgut and salivary gland cells, where it could be associated with fibronectin.

RPL5, RPL7a and RPL8 were found to bind to RSV particles in vitro and interact with RSV-NCP in YTHS. R-proteins are a major component of ribosomes that catalyze protein biosynthesis in the cytoplasm. Multiple lines of evidence demonstrate that r-proteins are not merely scaffolds needed to maintain the structure of mature ribosomes, and some r-proteins appear to possess regulatory functions in fundamental processes related to the cell cycle, apoptosis, development and oncogenes [36]–[38].

Currently, limited information is available on the functional relationship between r-proteins and virus infection. A genome-wide screen of *Drosophila melanogaster* genes demonstrates the critical role of r-proteins in virus accumulation [39]. *S. cerevisiae rpl5* mutants show defects in the ability to propagate endogenous M_1_ killer virus [40]. In studies related to plant DNA viruses, the P6 protein of Cauliflower mosaic virus (CaMV) is found in a complex with r-proteins. CaMV P6 interacts with about a dozen proteins from a ribosomal fraction, including r-proteins RPL18, RPL24, and RPL13, as well as the translation initiation factor eIF3 [41]. It is proposed that these interactions may be involved in the re-initiation of translation of polycistronic CaMV RNAs although the functional significance is yet to be determined. In the case of geminiviruses, RPL10a and RPL18ab are found to interact with nuclear shuttle protein interacting kinase (NIK), which is a virulence target of the begomovirus nuclear shuttle protein. *Rpl10a* loss-of-function mutants are more susceptible to an attenuated begomovirus, suggesting that RPL10a may be a component of the antiviral defense pathway mediated by NIK [42]. In regard to plant RNA viruses, large groups of r-protein mRNA transcripts are observed to be up-regulated in response to Turnip mosaic virus (TuMV, *potyvirus*) in *Arabidopsis thaliana*, Plum pox virus (PPV, *potyvirus*) and Tobacco mosaic virus (TMV, *tobamovirus*) in *Nicotiana benthamiana* [43]–[45]. The increased expression of suites of r-protein genes in response to virus infection is consistent with their co-regulated expression across a variety of other conditions [46]. It is an interesting phenomenon that cap-independent TuMV and Tomato bushy stunt virus (TBSV, *tombusvirus*) require RPS6 for their accumulation, whereas accumulation of TMV with the 5′ m^7^Gppp cap and the Ω leader is independent of RPS6 in *N. benthamiana*. However, the accumulation of both TuMV and TMV is dependent on RPL19, RPL13, RPL7, and RPS2 regardless of their translation strategies [43]. These results suggest potential roles for host r-proteins in modulating virus infection and accumulation.

It is demonstrated that the NCP of Sin nombre hantavirus (SNV, *Bunyaviridae*) can replace the entire cellular eIF4F complex to mediate directly viral mRNA translation initiation through interacting with mRNA cap and the 43S pre-initiation complex. SNV-NCP can bind to 40S ribosomal subunit and unphosphorylated eIF2, which are major functional components of the 43S complex [47]. In our experiments, *L. striatellus* r-proteins RPL5, RPL7a and RPL8 interacted with RSV-NCP. We suggested that three r-proteins played potential crucial roles in RSV infection and propagation in vector cells. The true biological significance of interactions between RSV and r-proteins remained to be elucidated.

Moreover, it was remarkable that interaction between the symbionin (*Buchnera* GroEL) and RSV particles was not found, whereas such an interaction was certainly expected to protect virus particles in insect haemocoel. The symbionin, from endosymbiotic bacteria *Buchnera aphidicola*, probably plays a crucial role in virus transmission by preventing virus particles from being proteolytically degraded in the insect haemocoel or by favoring the transport of the viral particles to the ASG of vector [17], although it is not involved in vector specificity [48], [49]. So far, the *Buchnera* GroEL gene from *L. striatellus* had not been cloned, and whether symbionin exists in *L. striatellus* remained to be further elucidated.

Although the true receptor for RSV had not yet been identified, our results suggested that three ribosomal proteins (RPL5, RPL7a and RPL8) might play potential crucial roles in the infection and propagation of RSV in vector cells. We also hypothesized that RACK and GAPDH3 were probably involved in the transcytosis mechanism of RSV particles in epithelial cells. It was obvious that the proteins identified in this study represented only a fraction of *L. striatellus* components that could interact with RSV during viral life cycle. Nevertheless, the current investigation identified five novel and unexpected insect proteins that bind to RSV particles, which should contribute to an ultimate understanding of molecular mechanisms on RSV's circulative-propagative transmission in *L. striatellus*. Transmission mechanisms of RSV remained to be further elucidated in future.

## Materials and Methods

### Preparation of virus and antibodies

RSV-infected rice samples were collected from rice fields in Jiangsu Province, China. RSV particles were purified from leaf material according to Xie [8]. Purified virus particles were characterized by 12% SDS-PAGE, and the virus concentration was determined using the Bradford assay [50] with bovine serum albumin (BSA) as a standard. Purified particles were stored at −70°C. Monoclonal antibodies (MAbs) against RSV were prepared by Zhejiang University and author's laboratory, and the titre of MAbs (3B9) was 1∶5 120 000 tested by indirect ELISA [2].

### Insects rearing

*L. striatellus* used in this study was collected from Jiangsu Province, China, and has been maintained in the laboratory for nearly 6 years. High-affinity and non-viruliferous populations were screened and reared in glass beakers as stock population. Rice plants (cultivar Wuyujing No. 3) as planthoppers' diet were grown in soil at 25°C with a photoperiod of 16 h/8 h (light/dark) in a growth incubator. After insects was introduced into a glass beakers which contained rice seedlings (2–3 cm high), the beakers were enclosed with a piece of nylon mesh. The planthoppers were transferred to the fresh seedling every 10–14 days to assure sufficient nutrition.

### Protein extraction

Approximately 200 insect adults above-mentioned were used for protein extraction. The insects were anesthetized with aether and frozen with liquid nitrogen in centrifuge tube. Protein extraction was performed as described by Nguyen *et al.* [51]. Briefly, total proteins were precipitated for overnight at −20°C with 10% trichloracetic acid (TCA) in acetone containing 0.07% (w/v) 2-mercaptoethanol, and then solubilized in an electrophoretic sample buffer consisting of 7 M urea, 2 M thiourea, 4% (w/v) CHAPS, 0.5% (v/v) IPG buffer pH 3–10, and 40 mM dithiothreitol (DTT). The sample was characterized by 12% SDS-PAGE, and protein concentration was determined according to Bradford [50], with BSA as standard protein. Final sample volume was adjusted to standardize the amount of protein (100 µg/100 µl) for electrophoresis.

### Two-dimensional electrophoresis (2-DE)

2-DE was performed according to Bio-Rad 2-DE Instruction Manual. For the first, isoelectric focusing (IEF) step, samples (300 µl/strip) were loaded on ReadyStrip IPG strips (11 cm) with non-linear pH 3–10 gradients, and resolved using the IPGphor apparatus (Bio-Rad). After active rehydration (50 V) for 14 h, IEF was performed following a voltage step-gradient (250 V Linear for 1 h, 1000 V Rapid for 1 h, 8000 V Linear for 4 h, and 8000 V Rapid for 40,000 V·hr) at 20°C, with a maximum current of 50 µA/strip. Before the second step (SDS-PAGE), the IPG strips were first equilibrated for 15 min in a solution containing 6 M urea, 0.375 M Tris-HCl pH 8.8, 2% (w/v) SDS, 20% (v/v) glycerol, and 2% (w/v) DTT, and then for 15 min in the same solution, substituting DTT with 2.5% (w/v) iodoacetamide. The second dimension was carried out in 1 mm-thick 12% SDS-polyacrylamide gels. Gels were run at 30 mA of constant current until the bromphenol blue dye front migrated 2 cm from the bottom.

### Western blot and virus overlay assay (Far-Western blot)

After electrophoresis, proteins in one gel were transferred onto a polyvinylidene difluoride (PVDF) membrane (Millipore) by a Bio-Rad semidry blotting procedure (20 V, 2.5 mA/cm^2^ for 45 min). To locate potential virus-binding protein pots from 2-DE gels for identification, the other gel was stained with Coomassie brilliant blue G-250.

The far-Western blot was performed as described by Kikkert *et al.* [12], with some modifications. The membrane was washed twice for 5 min each in PBST (137 mM NaCl, 2 mM KCl, 10 mM Na_2_HPO_4_, 2 mM KH_2_PO_4_, pH7.5, 0.05%Tween-20), and then blocked for 2 h at room temperature in blocking buffer (PBST, 5% Elk [skim instant milk]). The membrane was subsequently incubated overnight in blocking buffer containing purified RSV particles (5 µg/ml). After washed three times for 10 min each in PBST, membrane was incubated with MAbs against RSV (1∶3000 dilution) in blocking buffer for 3 h at 37°C. After washing as previously, antigen-antibody complexes were detected with a horseradish peroxidase (HRP)-conjugated goat anti-mouse secondary antibody (1∶10000 dilution) in blocking buffer for 1.5 h. After another series of washes, immobilized conjugates on the membrane were visualized by HRP substrate solution. To select spots from 2-DE gels for identification, parallel experiments were carried out.

### Image analysis and protein identification

The stained electrophoretic profiles of *L. striatellus* proteins were analyzed using the Melanie computer program (Bio-Rad). Comparisons between the stained 2-DE profiles in the gel and results of far-Western blot experiments on the membrane allowed the unambiguous selection of protein spots from 2-DE gels for Nano LC-ESI-CID-MS/MS analysis.

Selected protein spots were cut out from gels using a pipette tip manually, washed two times in 250 µl of 50% (v/v) acetonitrile for 15 min and then destained with 100 mM NH_4_HCO_3_ in 50% (v/v) acetonitrile for 15 min. After shrinking the gel pieces with acetonitrile, the proteins were reduced with 10 mM DTT in 100 mM NH_4_HCO_3_ (56°C, 1 h). After cooling to room temperature, the DTT solution was replaced with the same volume of 55 mM iodoacetamide in 100 mM NH_4_HCO_3_ (in the dark, 30 min). After alkylation, the gel pieces were washed with 100 µl 100 mM NH_4_HCO_3_ in 50% (v/v) acetonitrile for 10 min. The liquid phase was removed, and the gel pieces were completely dried in a vacuum centrifuge, followed by swelling in a digestion buffer containing 50 mM NH_4_HCO_3_, 5 mM CaCl_2_, and 20 ng/µl trypsin (Promega) in an ice-cold bath. After 45 min, the supernatant was removed and replaced with 10 µl the same buffer, but without trypsin, to keep the gel pieces wet during enzymatic cleavage (37°C, overnight). The digested peptides were extracted from gel slices with 5% (v/v) formic acid in 50% (v/v) acetonitrile.

The Agilent 1100 HPLC system was connected to an LTQ Orbitrap linear ion trap mass spectrometer (Thermo Fisher Scientific Inc., Waltham, MA). The instrument was equipped with a 20 mm×100 µm ID Aqua C_18_ trap column (Phenomenex, Torrance, CA), and a 200 mm×50 µm ID Reprosil C_18_ RP analytical column (Dr. Maisch, Ammerbuch-Entringen, Germany). Mobile phase A (solvent A) consisted of 0.1% (v/v) formic acid in water and mobile phase B (solvent B) of 0.1% (v/v) formic acid in acetonitrile. The tryptic peptides were separated by using a flow rate of 200 nl/min linear gradient from 0 to 60% solvent B over 90 min, followed by a 95% solvent B over 5 min. The re-equilibration of the column was done during 20 min by 100% of solvent A. The MS was operated in positive ion mode, and parent ions were isolated for fragmentation in data-dependent mode.

Raw data processing was done with Bioworks Browser software, version 3.3.1. The peptide sequences obtained from Nano LC-ESI-CID-MS/MS were searched against the protein sequences from NCBInr metazoa and other metazoa entries, using the Mascot algorithm (http://www.matrixscience.com). The search parameters were set as follows: enzyme, trypsin; fixed modifications, carbamidomethyl (C); variable modifications, oxidation (M); max missed cleavages, one; mass tolerances for MS/MS were 10 ppm and 0.5 Da. Protein identification was accepted when the matching scores were significant at P<0.05, as based on the Mowse score (Matrix Science, London, UK).

### Cloning and sequencing ORFs of virus-binding proteins

The peptide sequence tag analysis was performed to confirm the assigned proteins by searching peptide sequences against a transcriptome database of *L. striatellus* with the tBlastn tool. The related information of *L. striatellus* transcriptome database was provided by Prof. RX Fang and XY Chen (Institute of Microbiology, Chinese Academy of Sciences) [52]. After confirming proteins, contigs were obtained. Five pairs of primers for specific amplification of virus-binding proteins' genes were designed according to respective contig sequences (Table 3). Total RNA of insect was isolated from whole bodies, following the standard protocol of TRIzol reagent (Invitrogen). RT-PCR was performed by using oligo(dT) to prime first cDNA strand synthesis and five pairs of specific primers for subsequent PCR amplification using *Pfu* DNA polymerase. The amplified products were cloned individually using the pMD18-T vector system (TaKaRa) and sequenced using an automated dye terminator sequencing system (model 377; PE Applied Biosystems, Foster City, CA) according to the manufacturer's protocol. Full-length ORFs of five virus-binding proteins were analyzed and confirmed with DNAstar software.

**Table 3:** Sequences and restriction sites of PCR primers.

Primer and purpose	Sequence (5′→3′)	Modification
Construction for sequence and YTHS		
RACK-F	GCcatatgATGTCGGAAACTTTTGAT	Nde I
RACK-R	CAgaattcTTATCGTGAGACAGCAAC	EcoR I
RPL5-F	CGcatatgATGGGTTTCGTCAAAG	Nde I
RPL5-R	TAgaattcTCACGATTCGGCGTCG	EcoR I
GAPDH3-F	CGcatatgATGTCAAACATTGGAAT	Nde I
GAPDH3-R	GCggatccTTAATCTTTGGTTTGGAT	BamH I
RPL7a-F	GGcatatgATGGTGCAGAAGAAGC	Nde I
RPL7a-R	ATggatccCTAGCCCTGTTTCTGG	BamH I
RPL8-F	ACgaattcATGGGTAGGGTTATCCGCG	EcoR I
RPL8-R	CAggatccCTAATCGTCTCCCTTCTT	BamH I
NCP-F	CGcatatgATGGGTACCAACAAGCC	Nde I
NCP-R	CTggatccCTAGTCATCTGCACCTT	BamH I
Construction of expression vectors		
RACK-EcoR-F	CGgaattcATGTCGGAAACTTTTGAT	EcoR I
RACK-Hin-R	TGaagcttTTATCGTGAGACAGCAAC	Hind III
RPL5-Bam-F	CGggatccATGGGTTTCGTCAAAGT	BamH I
RPL5-R	TAgaattcTCACGATTCGGCGTCG	EcoR I
GAPDH3-Bam-F	GCggatccATGTCAAACATTGGAATT	BamH I
GAPDH3-Hin-R	CGaagcttTTAATCTTTGGTTTGG	Hind III
RPL7a-Bam-F	ACggatccATGGTGCAGAAGAAGCC	BamH I
RPL7a-Hin-R	CGaagcttCTAGCCCTGTTTCTG	Hind III
RPL8-F	ACgaattcATGGGTAGGGTTATCCGCG	EcoR I
RPL8-Hin-R	GCaagcttCTAATCGTCTCCCTT	Hind III

Lowercase indicates a restriction enzyme site.

### Yeast two-hybrid screen (YTHS)

To test the ability of virus-binding proteins to interact with RSV-NCP in the yeast two-hybrid system, a yeast two-hybrid screen (YTHS) was performed. The ORFs of virus-binding proteins were inserted individually in the yeast expression vector pGBKT7. The full-length coding sequence of RSV-NCP was amplified by RT-PCR, and then cloned into the yeast expression vector pGADT7 to produce pGAD-NCP. All constructs were sequenced to confirm their authenticity. Five sets of plasmids (pGBK-RACK/pGAD-NCP, pGBK-RPL5/pGAD-NCP, pGBK-GAPDH3/pGAD-NCP, pGBK-RPL7a/pGAD-NCP, and pGBK-RPL8/pGAD-NCP) were cotransformed respectively into *Saccharomyces cerevisiae* AH109 cells (Clontech) according to the Yeastmaker™ Yeast Transformation System 2 User Manual (Clontech). At the same time, two sets of plasmids, pGBKT7-53/pGADT7-RecT and pGBKT7-Lam/pGADT7-RecT (Clontech), were respectively cotransformed into *S. cerevisiae* AH109 cells, and used as positive control and negative control.

### Binding of expressed *L. striatellus* proteins to RSV particles in vitro

ORFs of five virus-binding proteins were amplified using *Pfu* DNA polymerase and specific primer pairs (Table 3), and cloned individually into a prokaryotic expression vector, pET-32a (Novagen). The recombinant plasmids, which were designated pET-RACK, pET-RPL5, pET-GAPDH3, pET-RPL7a and pET-RPL8, were sequenced to confirm their authenticity, and then transformed respectively into *E. coli* strain BL21 (DE3) pLys S (Novagen). The RACK, RPL5, GAPDH3, RPL7a and RPL8 genes of *L. striatellus* were expressed in *E. coli* BL21 (DE3) pLys S cells via IPTG inducing. After SDS-PAGE analysis of expression products, western blot was performed as described above. Blots of proteins were renatured essentially at room temperature as described by Kikkert *et al.* [12], with some modifications. The membrane was washed three times for 10 min each in binding buffer (25 mM Tris-HCl, pH 7.5, 50 mM NaCl, 2 mM DTT, 2 mM EDTA, 0.3% BSA, and 0.025% Nonidet P-40). Subsequently, the membrane was incubated for 45 min in denaturing buffer (7 M guanidine hydrochloride, 50 mM Tris-HCl, pH 8.0, 2 mM DTT, 2 mM EDTA, and 0.3% BSA). Membrane was washed four times for 5 min each in renaturation buffer (25 mM Tris-HCl, pH 7.5, 50 mM NaCl, 2 mM DTT, and 2 mM EDTA) followed by overnight incubation in the same buffer. After renaturation of blots, virus overlay assay was performed as described above. In order to avoid SDS-PAGE denaturation of protein samples, a dot immunobinding assay (DIBA) was modified to test binding between five *L. striatellus* proteins and virus. Expression products were dotted individually onto a nitrocellulose membrane (Pall), and proteins of wild type *E. coli* BL21 and *E. coli* BL21 (transformed pET-32a vector) were used as negative control. After air drying the membrane, overlay analysis with RSV particles was performed as described above.
